# Better care for people with diabetes and endocrine diseases

**DOI:** 10.1186/s40842-015-0003-7

**Published:** 2015-06-04

**Authors:** Meng H. Tan, Peter Arvan

**Affiliations:** Division of Metabolism, Endocrinology & Diabetes, Department of Internal Medicine, University of Michigan, Ann Arbor, MI 48104 USA

With this issue, we launch *Clinical Diabetes and Endocrinology* (CDE), a new journal for health researchers and professionals involved in the care of people with diabetes and endocrine diseases. CDE is published collaboratively by the University of Michigan and our partners at BioMed Central. Our vision is to share new research and clinical knowledge in diabetes and endocrine diseases with the health professional community, so that ultimately, our patients can have better care.

Each year, new knowledge and treatments for diabetes and endocrine diseases are uncovered in many parts of the world. In diabetes, discovery and development of the 11 classes of anti-hyperglycemia medications available today has spanned over nine decades [Table 1].

**Table 1:** Classes of anti-hyperglycemia medications

Class of drug	Year first approved in US
1. Insulin	
•Animal insulins	1923
•Human insulins	1982
•Insulin analogs	1996
2. Sulfonylureas	1955
3. Biguanide-Metformin	1995 (1957 in Europe)
4. Alpha glucosidase inhibitors	1995 (1990 in Germany)
5. Amylin analog	1995
6. Thiazolidinediones	1997
7. Glucagon-like peptide receptor agonists	2005
8. Dipeptidyl-peptidase inhibitors	2006
9. Bile acid sequestrant-Colesevelam	2008
10. Dopamine agonist-Bromocryptine	2009
11. Sodium-glucose co-transporter 2 inhibitors	2013

These medications have helped to improve glycemic control, but we need continued advances in understanding diabetes so that the burden of disease can be decreased both for individual patients and for society as a whole.

From idea to patient, translational research spans the spectrum from basic science, to clinical research, to clinical trials, to approval by regulatory agencies. Because as many as 387 million patients worldwide have been diagnosed with diabetes in 2013 [1], we need progress in all of these areas.

Among other endocrine disorders, genomics will unquestionably enhance our understanding and management of disease. The incidence of thyroid cancer has been increasing steadily [2]. In 2014, it was estimated there were 62,980 new cases of thyroid cancer in the US alone. New knowledge in molecular diagnosis [3] of thyroid cancer may greatly influence the choice of treatments and would benefit those affected. In adrenocortical carcinoma, genomic studies have allowed us to distinguish two groups of patients–one with poor prognosis and another with a far more favorable prognosis [4]. Translational research from mouse to human studies may lead to new ways to treat metastatic thyroid cancer [5].

To have a global impact, new knowledge should be readily available and freely accessible to health professionals worldwide. Traditionally, this new knowledge has been shared in print subscription journals, but the internet and digital publishing has revolutionized this. The seed for Open Access publications was planted in 1945 [6], but after a rather long germination period, Open Access publishing is now growing in leaps and bounds. Between 2000 and 2009 the number of Open Access journals increased from 740 to 4,796 and their combined Open Access articles increased from 19,500 to 191,850 [7]. This has been stimulated by the three calls to action (Budapest Declaration in 2002, Bethesda Statement in 2003, and Berlin Declaration in 2003) on Open Access publishing, which all advocated that “peer-reviewed research articles, donated for publication by authors with no expectation of compensation, should be available online, free, and with smallest possible number of usage restrictions” [6]. Indeed, between 2008 and 2014, the number of journals in PubMed Central with immediate free access has increased from 375 to 1,358 [8]. Consistent with this mandate, CDE is an immediate Open Access, peer-reviewed, high quality, online scholarly journal with a focus on clinical diabetes and endocrinology. As the journal matures, we expect that papers published in CDE will be indexed in PubMed and other key literature indices, and when the time comes, we will register with Thomson Reuters for the journal’s impact factor.

We plan to have a strong international presence and are already receiving queries from developing as well as developed nations around the world. With this in mind, our distinguished international editorial board comprises of experts from 19 nations and five continents. Our authors and readers will be health professionals who wish to share new knowledge that may lead to better understanding of diabetes and endocrine diseases. With this in mind, in our first issue, Hahr and Molitch describe clear, concise and simple guidelines for managing hyperglycemia in patients with diabetes complicated by chronic kidney failure [9]. These will show health professionals how to safely use anti-hyperglycemia medications to control hyperglycemia in this large group of patients. In addition, Minanni et al. from Brazil describe their rare challenging case of fatal factitious Cushing Syndrome as an example of Münchhausen’s Syndrome [10]. It took a team of clinicians and laboratory scientists to solve this case.

Our papers will cover clinical, translational, health care, epidemiology and healthy policy research. They will include original research, reviews, selective case reports, clinical trials, clinical practice guidelines, commentaries, editorials and others. Together, we aim to promote better care for people with diabetes and endocrine diseases globally.
